# Novel Antimicrobial Agents Based on Zinc-Doped Hydroxyapatite Loaded with Tetracycline

**DOI:** 10.3390/antibiotics13090803

**Published:** 2024-08-25

**Authors:** Simona Liliana Iconaru, Daniela Predoi, Carmen Steluta Ciobanu, Catalin Constantin Negrila, Roxana Trusca, Steinar Raaen, Krzysztof Rokosz, Liliana Ghegoiu, Monica Luminita Badea, Carmen Cimpeanu

**Affiliations:** 1 National Institute of Materials Physics, Atomistilor Street, No. 405A, 077125 Magurele, Romania; ciobanucs@gmail.com (C.S.C.); catalin.negrila@infim.ro (C.C.N.); ghegoiuliliana@gmail.com (L.G.); 2 National Centre for Micro and Nanomaterials, University Politehnica of Bucharest, 060042 Bucharest, Romania; truscaroxana@yahoo.com; 3 Department of Physics, Norwegian University of Science and Technology (NTNU), Realfagbygget E3-124 Høgskoleringen 5, NO 7491 Trondheim, Norway; steinar.raaen@ntnu.no; 4 Faculty of Electronics and Computer Science, Koszalin University of Technology, Śniadeckich 2, PL 75-453 Koszalin, Poland; rokosz@tu.koszalin.pl; 5 Faculty of Horticulture, University of Agronomic Sciences and Veterinary Medicine, 59 Marasti Boulevard, 011464 Bucharest, Romania; badea.artemisia@gmail.com; 6 Faculty of Land Reclamation and Environmental Engineering, University of Agronomic Sciences and Veterinary Medicine of Bucharest, 59 Marasti Boulevard, 011464 Bucharest, Romania; carmencimpeanu@yahoo.com

**Keywords:** zinc, hydroxyapatite, tetracycline, in vitro biological studies, composition, biomedical applications

## Abstract

In this paper, we present for the first time the development of zinc-doped hydroxyapatite enriched with tetracycline (ZnHApTe) powders and provide a comprehensive evaluation of their physico-chemical and biological properties. Various techniques such as X-ray diffraction (XRD), X-ray photoelectron spectroscopy (XPS), scanning electron microscopy (SEM), and Fourier transform infrared spectroscopy (FTIR) were used for the sample’s complex evaluation. Moreover, the biocompatibility of zinc-doped hydroxyapatite (ZnHAp) and ZnHApTe nanoparticles was evaluated with the aid of human fetal osteoblastic cells (hFOB 1.19 cell line). The results of the biological assays suggested that these nanoparticles hold great promise as potential candidates for the future development of novel biocompatible and antimicrobial agents for biomedical applications. The antimicrobial properties of the ZnHAp and ZnHApTe nanoparticles were assessed using the standard reference microbial strains *Staphylococcus aureus* ATCC 25923, *Escherichia coli* ATCC 25922, and *Candida albicans* ATCC 10231. The results of the in vitro antimicrobial assay demonstrated that both tested materials exhibited good antimicrobial activity. Additionally, these data also indicated that the antimicrobial effects of the ZnHAp nanoparticles were intensified by the presence of tetracycline (Te). Furthermore, the results also suggested that the antimicrobial activity of the samples increased with the incubation time.

## 1. Introduction

Currently, the prevention of infections and their management (for example, those associated with orthopedic and dental implant procedures/operations) still represent a major challenge for both the medical and the scientific community. The standard treatment for these infections usually involves the systemic administration of antibiotics at high doses for long time periods that come with several important disadvantages (such as high costs and toxicity) [1,2]. On the other hand, the increasing microbial resistance represents a global health problem that requires the funding of new and alternative antimicrobial agents [3]. The calcium phosphate compounds, specifically hydroxyapatite (HAp), are known as the main inorganic constituent of hard tissues from the human body, such as teeth and bones [4,5]. Synthetic biomaterials based on HAp have attracted the researcher’s attention mainly because of their close resemblance to the mineral composition of human bones [4,5]. Moreover, HAp possesses the ability to promote the formation of new bone tissue and exhibit excellent osteoconductive properties [4,5,6].

Furthermore, the hexagonal structure of stoichiometric hydroxyapatite (HAp) allows substitution with various ions such as zinc, silver, cerium, samarium, and magnesium [7,8,9,10,11]. Among them, zinc is a trace metal that can be found in abundance in bone tissue, enhances bone metabolism and formation, prevents bone loss, and increases bone density [12,13]. In addition, according to previous studies, zinc-doped biphasic calcium phosphate ceramics and zinc-doped HAp have shown excellent bone formation and superior bone-implant attachment in animal studies [14]. Additionally, zinc-containing apatite layers on titanium rods significantly increased the proliferation and differentiation of fibroblastic and osteoblastic cell lines [13].

In addition, zinc is recognized for its antimicrobial activity against various microbial agents, such as *Escherichia coli*, *Pseudomonas aeruginosa*, *Staphylococcus aureus*, *C. albicans*, etc. [15,16]. In the studies previously reported, it was shown that zinc concentration from zinc-doped hydroxyapatite colloids strongly influences their in vitro antimicrobial activity against *Escherichia coli* and *Staphylococcus aureus* [17]. Similar results regarding the antimicrobial activity of zinc-doped hydroxyapatite materials against *Staphylococcus aureus* and *Escherichia* sp. were also reported by Ofudje, E.A., et al. [3]. Moreover, their results highlight that the obtained zinc-doped HAp composites possess excellent bioactive activity [3].

The tetracycline class represents a type of bacteriostatic agent with a broad spectrum of antimicrobial activity. They are effective against both Gram-positive and Gram-negative bacteria, including aerobic and anaerobic types. In addition, tetracyclines are effective in treating infections caused by *Mycoplasma*, *Rickettsiae*, *Chlamydia*, certain protozoa, and spirochetes [18]. Therefore, the development of new biomaterials based on zinc, hydroxyapatite, and tetracycline could represent a proper alternative for the prevention of infection occurrence associated with bone/teeth surgeries/procedures. In a study carried out by Rusu, L.C. [19], the results obtained on new types of bone grafts with antimicrobial properties (obtained by combining hydroxyapatite with a carboxymethylcellulose-collagen gel) used to deliver tetracycline over a long period of time are presented. Their results showed that these grafts could be used to treat infected bone defects [19]. The new types of grafts offer efficient local administration of the antibiotic, which leads to the minimization of systemic side effects [19]. Another study conducted by Rivadeneira, J. et al. [20] reported the development of tetracycline hydrochloride was incorporated into collagen type I membranes coated with bioactive glass to prevent wound infections. The antibiotic was released over 72 h and showed antibacterial activity against *Staphylococcus aureus*. The incorporation of tetracycline was dependent on its initial concentration, but the efficacy in inhibiting bacterial growth was similar across different concentrations, indicating the composite’s potential in preventing wound infections [20]. The study reported by Soriano-Souza, C., and collaborators [21] evaluates the hydroxyapatite ceramic microspheres loaded with doxycycline (HADOX) from the physical, chemical, and biological points of view. Their results underline that HADOX microspheres effectively inhibited bacterial growth for up to 7 days and did not significantly affect osteoblast viability compared with non-loaded HA microspheres [21]. In rat socket healing (after tooth extraction) experiments, HADOX facilitated bone formation and controlled inflammation, suggesting its potential as a biomaterial for enhancing bone repair in infected sites [21]. The previous studies conducted by D. Predoi and coworkers [22] revealed that the excellent antimicrobial activity of tetracycline embedded in silver-doped hydroxyapatite suspensions depends on the incubation time [22].

The aim and novelty of this work mainly consist of the development of zinc-doped hydroxyapatite enriched with tetracycline powders for the first time and their complex evaluation from a physicochemical and biological point of view. The obtained powders were evaluated using techniques such as X-ray diffraction (XRD), X-ray photoelectron spectroscopy (XPS), scanning electron microscopy (SEM), and Fourier transform infrared spectroscopy (FTIR). The in vitro biological activity was analyzed using a hemolysis assay, an MTT assay, and a lactate dehydrogenase (LDH) release measurement. Moreover, the in vitro antimicrobial activity of nanopowders was assessed using common reference strains such as *Staphylococcus aureus* ATCC 25923, *Escherichia coli* ATCC 25922, and *Candida albicans* ATCC 10231.

## 2. Results and Discussions

**Figure 1:**
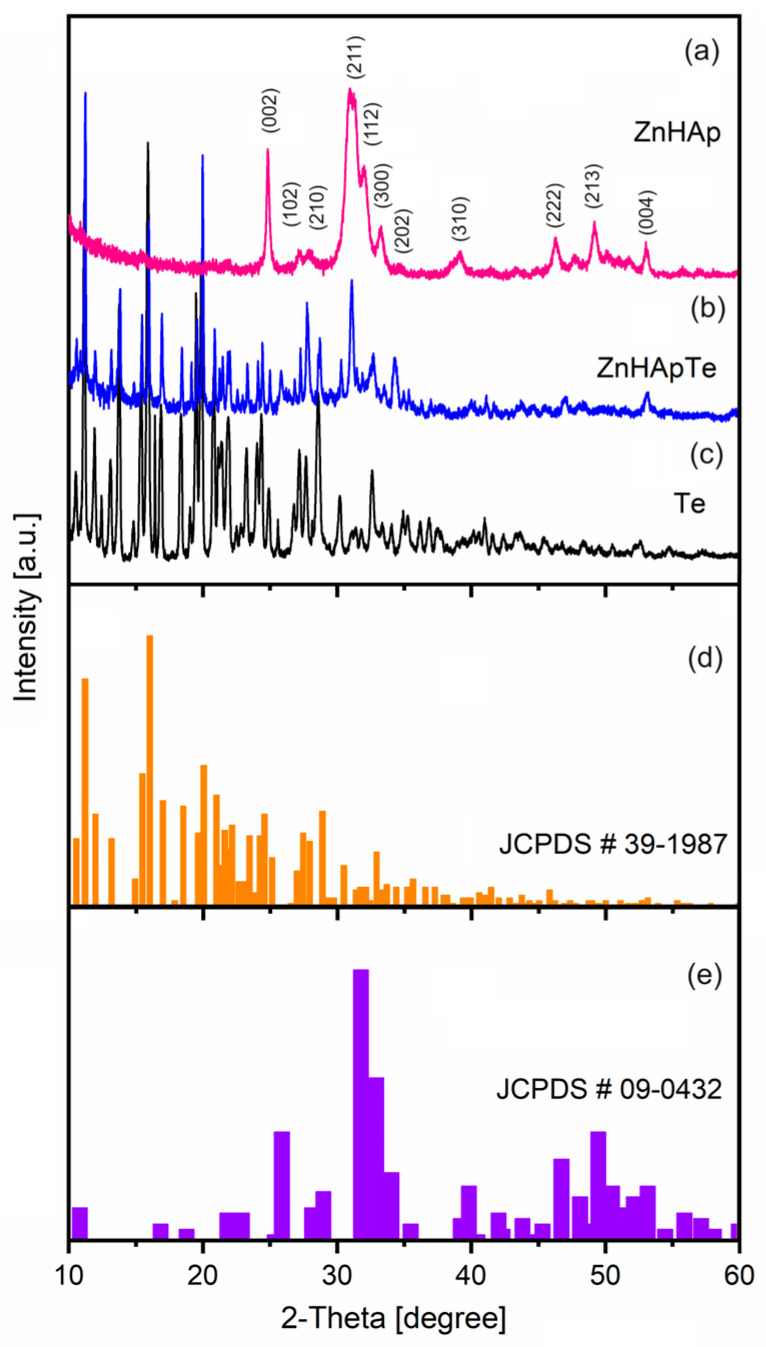
XRD patterns of the ZnHAp (**a**), ZnHApTe (**b**), and tetracycline (**c**) samples and standard database JCPDS #39-1987 (**d**) and JCPDS #09-0432 (**e**).

Figure 1 illustrates the typical XRD patterns of the ZnHAp (Figure 1a), ZnHApTe (Figure 1b), and Te (Figure 1c) samples. The standard database JCPDS #09-0432 (Figure 1e) and JCPDS #39-1987 (Figure 1d) of hydroxyapatite (HAp) and tetracycline (Te) were presented. The diffraction peaks observed in the XRD spectra of the ZnHAp sample correspond to the hexagonal (P63/m) lattice of HAp in agreement with the standard JCPDS database (JCPDS #09-0432). On the other hand, the diffraction peaks observed in the XRD spectra of the ZnHApTe sample (Figure 1b) correspond to the hexagonal lattice of HAp and tetracycline (Te) according with the standard database JCPDS #09-0432 and JCPDS #39-1987. The typical XRD peaks of the HAp structure were identified in Figure 1a. On the other hand, the HAp typical peaks were revealed in Figure 1b. Moreover, the typical peaks of Te were also observed in Figure 1b. Additionally, no zinc oxide, impurities, or secondary phases were detected. XRD analysis confirmed the obtained composite based on hydroxyapatite and tetracycline.

**Figure 2:**
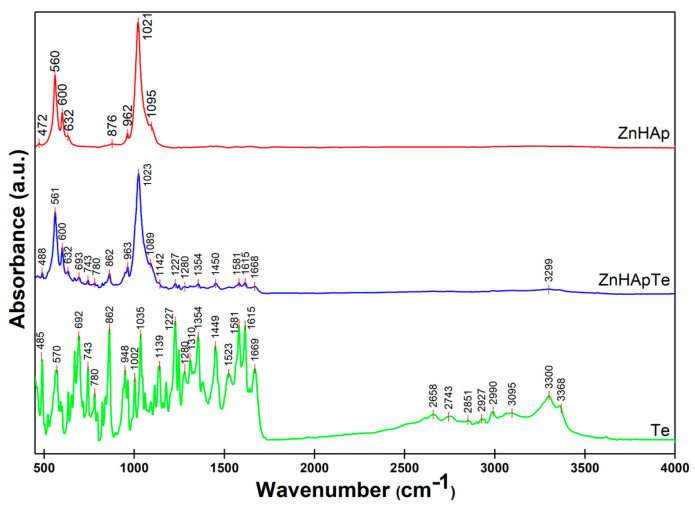
FTIR general spectra obtained for ZnHAp, ZnHApTe, and Te samples.

Figure 2 presents the FTIR general spectra obtained for ZnHAp, ZnHApTe, and Te samples. For the ZnHAp samples, the FTIR spectra reveal the presence of the vibrational maxima that are associated with the vibration of functional groups from the hydroxyapatite structure. Therefore, the vibrational maxima centered around 962 cm^−1^ is characteristic of the ν_1_ non-degenerate symmetric stretching mode of the P-O bond, indicating the presence of HAp in the studied sample [22]. The vibrational maxima centered around 472 cm^−1^ (ν_2_), 560 cm^−1^ (ν_4_), 600 cm^−1^ (ν_4_), 1021 cm^−1^ (ν_3_), and 1095 cm^−1^ (ν_3_) are characteristic of the vibration of the PO_4_^3−^ group [22,23]. The vibrational maxima centered approximately at 632 cm^−1^ is usually associated with the vibrational modes of structural OH^−^ groups [22]. Meanwhile, the vibrational maxima centered around 876 cm^−1^ appear because of the presence of carbonate groups in the ZnHAp sample [22].

In the FTIR spectra of tetracycline recorded between 450 and 4000 cm^−1^, the main vibrational maxima characteristic to the aromatic ring stretching vibrations (C=C) that are centered between 1449 cm^−1^ and 1669 cm^−1^ [22,23,24,25] are observed. In addition, in the FTIR spectra of tetracycline, the vibrational maxima characteristic of the aromatic deformation (=C-H) are present. These maxima are centered between 669 cm^−1^ and 948 cm^−1^ [22,25]. The vibrational maxima centered around 1354 cm^−1^ could be attributed to either the terminal dimethyl bending vibration mode, symmetric CH_3_ bending mode, or C-O stretching [22,24,25]. Additionally, the vibrational maxima centered at 1227 cm^−1^ and 1112 cm^−1^ are attributed to the C-N stretching vibration mode [22,24,25,26]. In addition, the vibrational maxima specific to the out-of-plane aromatic ring deformation are observed around 485 cm^−1^, 668 cm^−1^, and 692 cm^−1^ [22,25]. The vibrational maxima associated with the in-plane ring deformation are centered at 633 cm^−1^ [22,25].

Thus, the FTIR spectra of the ZnHApTe powders clearly highlight the presence of the main vibrational maxima characteristic to the functional groups from both hydroxyapatite and tetracycline molecular structures. The maxima observed in the FTIR spectra of the ZnHApTe powders are slightly shifted compared with the maxima observed in the reference FTIR spectra of the ZnHAp and Te samples. In addition, the additional vibrational maxima that appear in the ZnHApTe sample because of the presence of tetracycline are less intense compared with those in the reference spectra of the antibiotic. These results suggest that the antibiotic interacts well with the hydroxyapatite structure and are in good agreement with the results previously reported by D. Predoi et al. [22].

**Figure 3:**
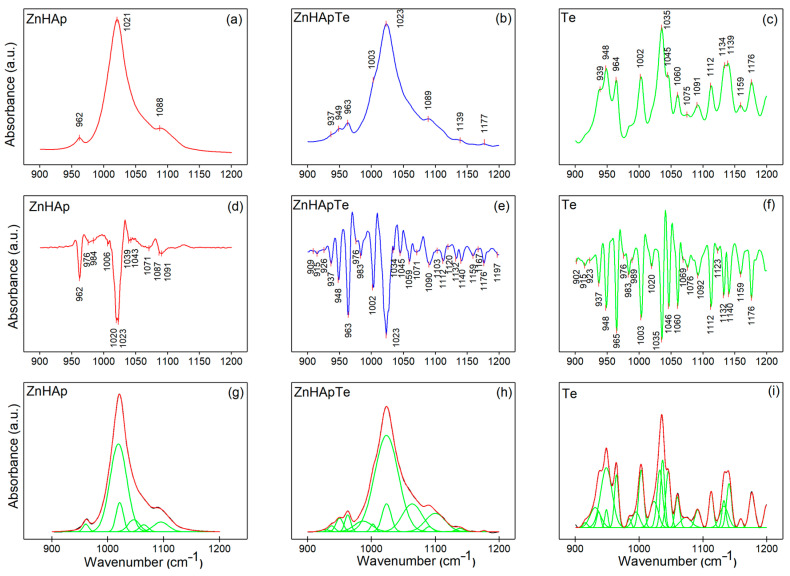
(**a**–**c**) FTIR spectra in the 900–1200 cm^−1^ spectral domain, (**d**–**f**) second derivative spectra, and (**g**–**i**) deconvoluted FTIR spectra of the ZnHAp, ZnHApTe, and Te samples.

To obtain valuable information regarding the subtle spectral changes in the ZnHAp, ZnHApTe, and Te samples, the FTIR spectra between 900 cm^−1^ and 1200 cm^−1^ were analyzed using second derivative and curve fitting methods (Figure 3). This spectral region is characteristic of the ν_1_ and ν_3_ vibration of PO_4_^3−^ (for the ZnHAp sample). The second derivative spectra and deconvoluted spectra of the Te sample are presented to show that the additional maxima that appear in the FTIR spectra of the ZnHApTe sample belong to Te. In the second derivative spectra of the ZnHAp (Figure 3d) sample, the ν_3_ main vibrational maxima of the phosphate group are centered around 1020 cm^−1^, 1023 cm^−1^, 1039 cm^−1^, 1043 cm^−1^, 1071 cm^−1^, and 1091 cm^−1^ [27,28]. Furthermore, the ν_1_ vibrational maxima of the phosphate group is centered around 962 cm^−1^ in Figure 3d. The second derivative spectra of the tetracycline (Figure 3f) reveal the presence of multiple vibrational maxima that are usually associated with the aromatic C-H in-plane deformation (between 1200 and 1000 cm^−1^) and to C-N stretching (around 965 cm^−1^) [29]. To better highlight the presence of both ZnHAp and Te in the ZnHApTe composition, FTIR second derivative analysis was conducted (Figure 3f). The obtained spectra revealed that the characteristic maxima of HAp and Te are slightly displaced compared with their position in the reference FTIR spectra. Thus, the vibrational maxima associated with the ν_3_ vibration of the phosphate group are centered at about 1023 cm^−1^, 1034 cm^−1^, 1045 cm^−1^, 1071 cm^−1^, and 1090 cm^−1^. The presence of HAp in the ZnHApTe was also confirmed by the presence in the second derivative spectra of the intense ν_1_ vibration band of the phosphate group at about 963 cm^−1^. The presence of the tetracycline in the ZnHApTe sample was confirmed by their specific aromatic C-H in-plane deformation and C-N stretching vibrational bands. It was noticed that the main functional groups that conduct the ZnHApTe absorbance in the studied spectral domain are assigned to the hydroxyapatite (namely phosphate groups) and tetracycline structure (C-H in-plane deformation and C-N stretching).

Figure 3g–i shows the experimental and calculated contours overlaid (red) along with the individual subbands (green) identified through curve fitting analysis of the ZnHAp, ZnHApTe, and Te samples in the 900–1200 cm^−1^ region. Thus, six components were needed to achieve a satisfactory fit for the ZnHAp sample (Figure 3g). The six subbands are centered at 1022 cm^−1^, 1019 cm^−1^, 1047 cm^−1^, 1095 cm^−1^, 961 cm^−1^, and 1065 cm^−1^. In the case of the ZnHapTe sample, a satisfactory fit was obtained with the aid of fourteen subbands. Moreover, twenty-three subbands were used to obtain a satisfactory fit in the case of the Te sample. Therefore, the results of the FTIR studies demonstrate the presence of both HAp and Te in the analyzed powders and their good interaction.

**Figure 4:**
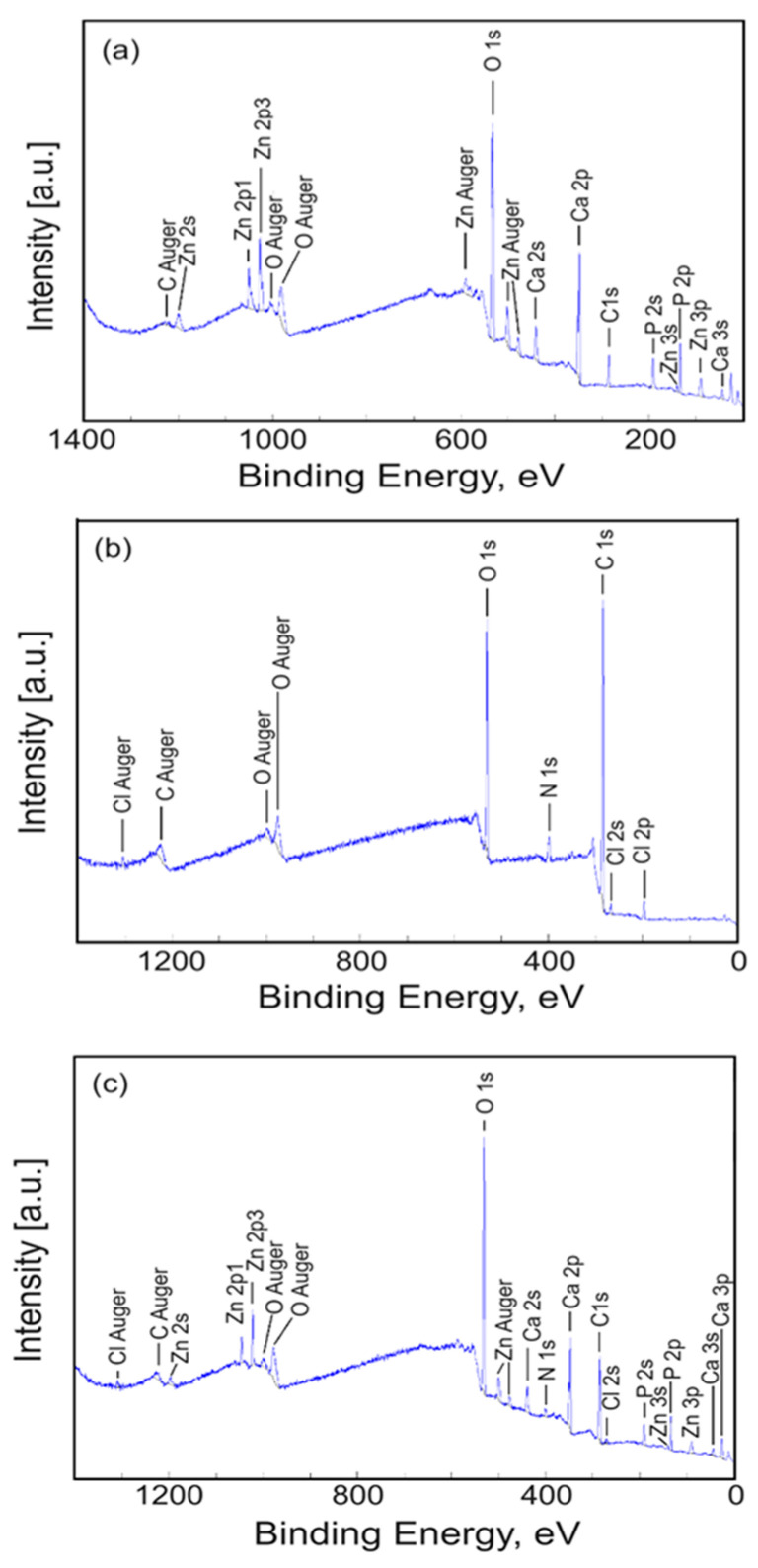
General XPS spectra of the ZnHAp (**a**), Te (**b**), and ZnHApTe (**c**) samples.

In order to analyze the surface chemistry and elemental composition of the ZnHAp, Te, and ZnHApTe samples, an XPS study was performed. The general XPS spectra of the samples are presented in Figure 4. The XPS studies highlighted the presence of zinc (Zn), oxygen (O1s), calcium (Ca2p), phosphorus (P2p), and carbon (C1s) in the XPS general spectra of the ZnHAp and ZnHApTe samples (Figure 4a,c). On the other hand, nitrogen (N1s) was observed in the XPS general spectra of the Te and ZnHApTe samples (Figure 4b,c). The presence of N 1s demonstrates the presence of tetracycline in the ZnHApTe sample. In addition, the presence of O1s and C1s also demonstrates the presence of tetracycline in the ZnHApTe sample. It can be observed that the intensities of O1s and C1s peaks are more intense in the case of the ZnHApTe sample (Figure 4c) than in the case of the ZnHAp (Figure 4a) sample. Moreover, the presence of chlorine (C1s) in the XPS spectra of the Te and ZnHApTe samples is due to the impurities present in the tetracycline powder.

**Figure 5:**
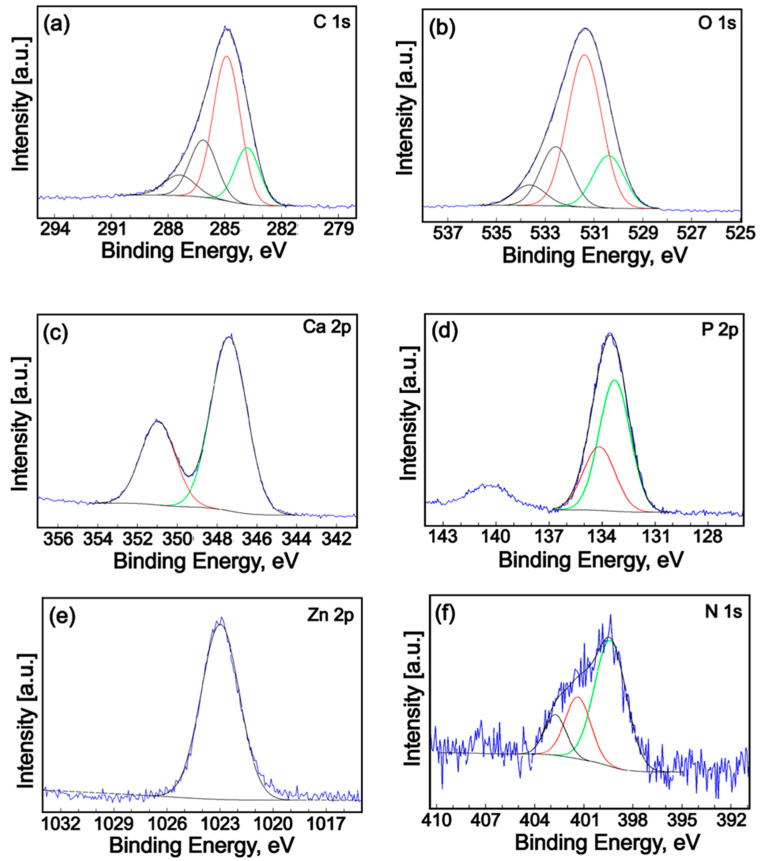
High-resolution XPS spectra and curve-fitting results of C1s (**a**), O1s (**b**), Ca2p (**c**), P2p (**d**), Zn2p (**e**) and N1s (**f**) for ZnHApTe sample.

XPS high-resolution spectra of constituent elements C1s, O1s, Ca2p, P2p, Zn2p, and N1s of the ZnHApTe sample are presented in Figure 5.

The high-resolution spectra of C1s of the ZnHApTe (Figure 5a) show a single C-C bond that was identified at a binding energy of 284.9 eV [30]. A peak of about 283.1 eV was also observed for C=C bonds (sp2 hybridization). The peak observed at 286.14 eV was associated with C-O single bonds. The peak at 287.4 eV allocated to C=C or O-C-O bonds was distinguished. High-resolution XPS spectra of O1s oxygen for the ZnHApTe sample are shown in Figure 5b. The four distinct signals at binding energy at 530.40, 531.31, 532.56, and 533.64 eV were detected. The signal at 530.40 eV was attributed to C=O double bonds with aromatic C. On the other hand, the signal at 530.40 eV can be associated with the bonding of oxygen with calcium (Ca) in agreement with Zhang et al. [31]. The signal identified at 531.31 eV can be ascribed to the Ca-O bond [32] of hydroxyapatite (HAp) and C=O double bonds with aliphatic C. Moreover, in prior studies [33,34,35], it was shown that the binding energy of chemisorbed oxygen species (O-) is in the range of 531.0–531.5 eV. The signal at 532.56 eV could be attributed to the P-O bond [32] as well as simple C-O bonds with aliphatic C. The signal detected at 533.64 eV can be assigned to O-H [36,37]. The high-resolution XPS spectrum of Ca2p (Figure 5c) shows two specific lines (2p3/2 and 2p1/2) spaced at approximately 3.6 eV with an area ratio close to 2:1. The binding energy of the two peaks (347.40 and 350.97 eV) is specific to hydroxyapatite [38]. In the high-resolution XPS spectrum of P2p, two specific lines (2p3/2 and 2p1/2) spaced at approximately 0.9 eV with an area ratio close to 2:1 were observed (Figure 5d). The binding energy of the two peaks (133.29 and 134.16 eV) is specific to hydroxyapatite [39,40]. In Figure 5e, the high-resolution XPS spectrum of Zn2p is presented. After deconvolution, the maximum peak of Zn2p3/2 was identified around the binding energy of approximately 1022.5 eV [41]. The obtained results are in good agreement with previously presented data [42]. Moreover, following the replacement of Ca^2+^ ions with Zn^2+^, the valence of zinc did not change. The full width also confirms this fact at half maximum (FWHM) of the 2p3/2 signal. The FWHM of the 2p3/2 signal was 2.54 eV, which is in agreement with previously obtained results [43]. High-resolution XPS spectra of the N1s peak of ZnHApTe are exhibited in Figure 5f. The N1s peak was deconvoluted in three components. The first component was located at 399.32 eV. The second component was identified at 401.35 eV. The third component was observed at 402.73 eV. The first component could be associated with C-N single bands with aromatic C. The second component located at 401 eV indicates a protonated N. The third component corresponds to the beginning of N-O bond formation.

**Figure 6:**
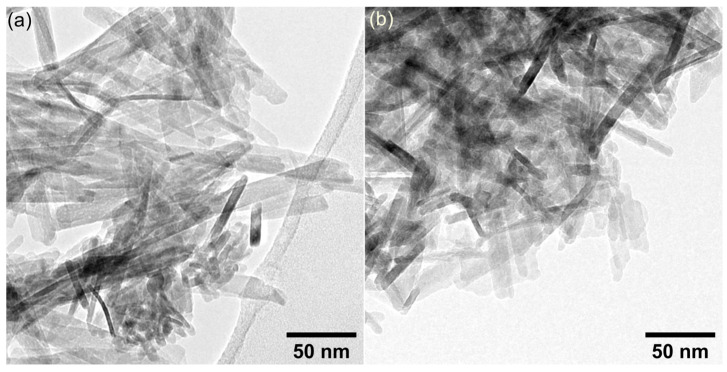
TEM images of ZnHAp (**a**) and ZnHApTe (**b**).

The transmission electron microscopy studies were used to obtain information about the morphology features of the ZnHAp and ZnHApTe nanoparticles. The results of the TEM studies are presented in Figure 6. As can be observed, both samples possess an acicular morphology. Moreover, the TEM studies also underline the nanometric dimension of the ZnHAp and ZnHApTe nanoparticles and their tendency to form agglomerates.

**Figure 7:**
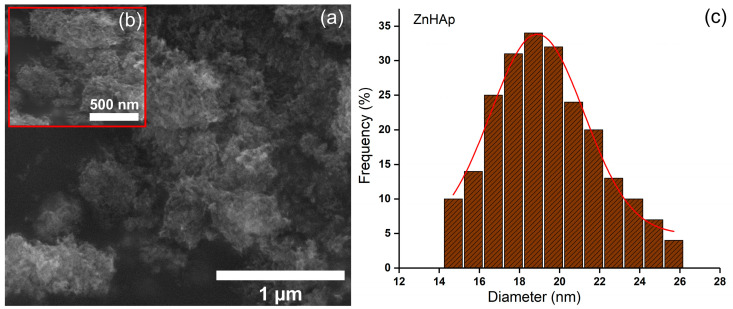
(**a**,**b**) SEM images and (**c**) particle size distribution obtained for the ZnHAp sample.

**Figure 8:**
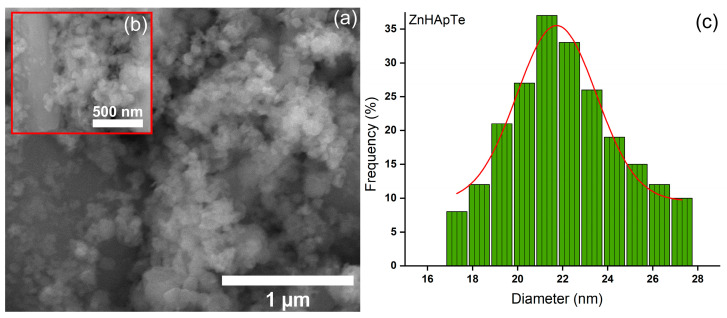
(**a**,**b**) SEM images and (**c**) particle size distribution obtained for the ZnHApTe sample.

Figure 7 and Figure 8 illustrate the SEM images and mean particle diameter of the ZnHAp and ZnHApTe nanoparticles. The SEM images presented in Figure 7 and Figure 8 are recorded at x100.000 (Figure 7a and Figure 8a) and x200.000 (inset of Figure 7b and Figure 8b). In the case of the ZnHAp nanoparticles, the SEM images reveal their nanometric dimension and acicular morphology. A slight change in the morphology was noticed for the ZnHApTe nanoparticles. The change in morphology could be attributed to the presence of Te in the sample. For both samples, the SEM images reveal their tendency to form agglomerates. The mean particle diameters determined by SEM analysis were 18.7 ± 2 nm for ZnHAp nanoparticles and 21.3 ± 2 nm for ZnHApTe nanoparticles.

**Figure 9:**
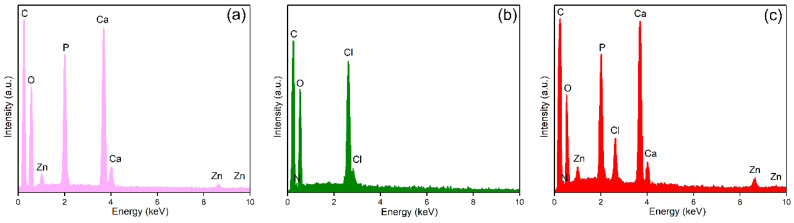
EDS spectra of the ZnHAp (**a**), Te (**b**), and ZnHApTe (**c**) samples.

Information about the chemical composition of the ZnHAp, ZnHApTe, and Te samples was obtained through EDS studies. The results of the EDS studies are illustrated in Figure 9.

The EDS spectra of the ZnHAp sample underline the presence of zinc (Zn), calcium (Ca), phosphorus (P), and oxygen (O). All these chemical elements belong to the chemical composition of zinc-doped hydroxyapatite nanoparticles. The EDS spectra of tetracycline reveal the presence of chlorine (Cl), nitrogen (N), and oxygen (O) in the sample. Furthermore, the EDS spectra of ZnHApTe highlight the presence of chemical elements from the ZnHAp and tetracycline samples. Thus, in Figure 9b, the presence of nitrogen (N), oxygen (O), calcium (Ca), chlorine (Cl), zinc (Zn), and phosphorus (P) is underlined. In all the EDS spectra, a carbon line could be observed because of the carbon tape on which the powders are placed in order to be analyzed. In the case of the Te and ZnHApTe samples, tetracycline also contributed to the C line in the EDS spectrum because of the carbon found in its chemical composition. The results of the EDS studies demonstrate that the analyzed samples are chemically pure based on the absence of additional lines in their EDS spectra.

Furthermore, the EDS quantitative analysis of the ZnHAp and ZnHApTe samples (x_Zn_ = 0.1; (Ca + Zn)/P = 1.67) was performed, and the results are presented in Table 1.

**Figure 10:**
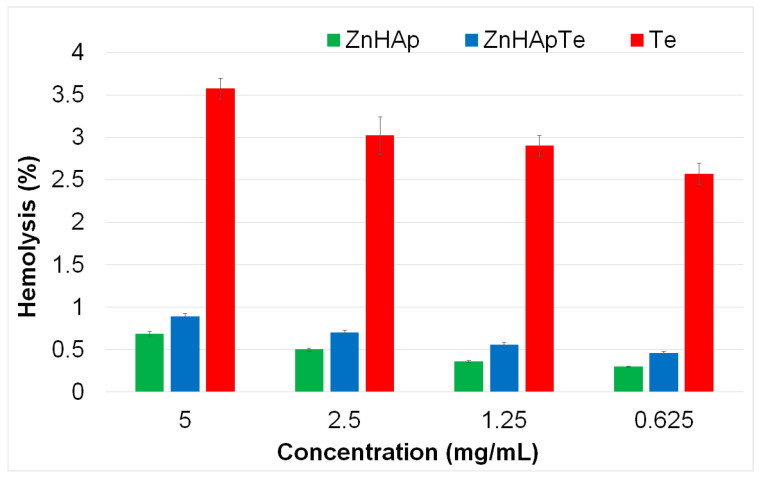
Percentage hemolysis of sheep red blood cells (RBCs) exposed to different concentrations of ZnHAp, ZnHApTe, and Te.

The cytotoxicity of the ZnHAp and ZnHApTe nanoparticles was assessed through hemocompatibility and biocompatibility studies. Hemolysis studies are usually used to evaluate the potential hemolytic activity of nanoparticles. The hemolysis assay provides important information about the potential cytotoxic effects of the tested substance. The hemolysis index is a significant parameter when assessing materials for their potential use in biomedical applications. The aim of this assay is to determine whether the tested substances can cause the rupture of red blood cells (RBCs), which can release hemoglobin into the bloodstream and lead to hemolysis. A low hemolytic index (<5%) indicates that the tested substances are hemocompatible and safe for use in biomedical applications, while a moderate hemolytic index (5–20%) usually signifies the need for further testing to ensure the safety usage of the substance. On the other hand, a high hemolytic index (>20%) indicates that the tested substances may not be safe for biomedical applications because of their significant hemolytic activity. Substances that exhibit a high hemolytic activity can cause damage to red blood cells, release hemoglobin, and provoke various negative physiological responses such as inflammation, thrombosis, and organ damage, rendering them unsuitable for biomedical applications. Contrarily, substances that exhibit a low hemolytic index are suitable for biomedical use because of their minimal risk of possible adverse reactions and their superior compatibility with biological systems. The results of the hemocompatibility assay in the case of the ZnHAp and ZnHApTe nanoparticles revealed that the hemolytic activity was below 1% for both tested samples. The results depicting the hemolytic index obtained for ZnHAp and ZnHApTe nanoparticles are presented graphically as mean ± SD in Figure 10.

The results of the hemolytic activity highlighted that none of the tested concentrations of ZnHAp, ZnHApTe, and Te caused hemolysis. In addition, the values obtained for the hemolytic index were well within the acceptable hemocompatibility limits for biomaterials. The results showed that the ZnHAp and ZnHApTe nanoparticles exhibited a hemolytic activity of less than 1%, while the hemolytic index of Te was above 3.5%. On the other hand, these data suggested that the hemolysis index increased with an increase in the concentration. Furthermore, the results showed that the ZnHAp nanoparticles exhibited a lower hemolytic index compared with ZnHApTe. These results could be attributed to the presence of tetracycline in the ZnHAp sample. These findings demonstrated that both ZnHAp and ZnHApTe nanoparticles showed a low hemolytic index, supporting the fact that they could be suitable for further cytotoxicity determinations to confirm their safety for usage in biomedical applications.

**Figure 11:**
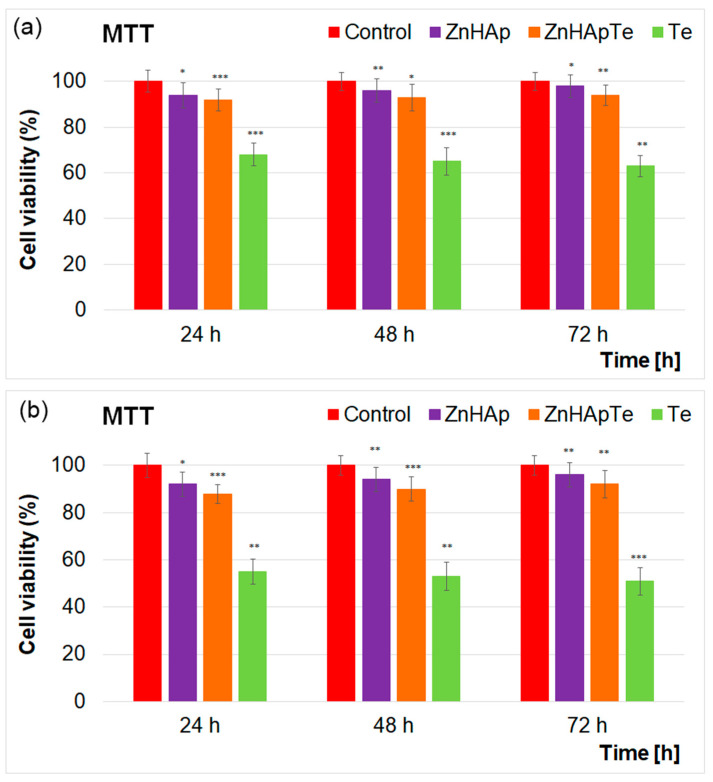
Cell viability of hFOB 1.19 cells incubated with 50 µg/mL (**a**) and 200 µg/mL (**b**) of ZnHAp, ZnHApTe, and Te for 24, 48 and 72 h. The results are represented as mean ± standard deviation (SD) and are expressed as percentages of control (100% viability). The statistical differences between untreated and treated groups were determined by ANOVA, and the results are significant at *p* < 0.05 (*); *p* < 0.01 (**); *p* < 0.001 (***).

Additional information regarding the cytotoxicity of the ZnHAp, ZnHApTe, and Te nanoparticles was obtained with the aid of the colorimetric MTT assay. For this purpose, the cell viability of hFOB 1.19 cells was evaluated after their exposure to different concentrations of the ZnHAp, ZnHApTe, and Te nanoparticles at three different time intervals. The results of the MTT assay are depicted in Figure 11a,b.

The results of the MTT assay depicted in Figure 11a,b revealed that the cell viability of the hFOB 1.19 cells remained above 92% after being exposed to 50 µg/mL ZnHAp and ZnHApTe nanoparticles for 24, 48, and 72 h. In addition, the results of the MTT studies highlighted that after 48 h and 72 h of exposure, the cell viability of the hFOB 1.19 cells exposed to ZnHAp increased, reaching 96% and 98%, respectively, which emphasizes that the ZnHAp nanoparticles exhibit good biocompatible properties towards hFOB 1.19 cells at a concentration of 50 µg/mL. On the other hand, for a concentration of 200 µg/mL, the cell viability presented a slight decrease for both the ZnHAp and ZnHApTe samples. The results of the MTT assays also highlighted that the Te sample exhibited the lowest degree of biocompatibility for both tested concentrations. The cell viability of hFOB 1.19 cells incubated with 50 and 200 µg/mL Te powders was equal and below 60% for both tested concentrations and for all the incubation periods. These results are in good concordance with other studies reported on the biological properties of zinc-doped hydroxyapatite biocomposites [44,45,46,47,48,49]. These findings indicated that the ZnHAp nanoparticles exhibited good biological properties when exposed to hFOB 1.19 cells. Studies have shown that ZnHAp supports the viability and proliferation of human osteoblast cells [44], which are essential for bone formation and repair. The presence of zinc ions could enhance the cell’s proliferation and differentiation. The results obtained for both the ZnHAp and ZnHApTe nanoparticles are in good agreement with the research of Tank et al., demonstrating the good biocompatibility of ZnHAp with human osteoblast cells (MG-63). Similar results were reported by Thian et al. [44] in their study regarding “*zinc-substituted hydroxyapatite: a biomaterial with enhanced bioactivity and antibacterial properties*”, which highlighted that ZnHAp exhibited very good biocompatible properties against mesenchymal stem cells (MSCs) derived from human adipose tissue. In addition, the biological properties of zinc-doped hydroxyapatite on the MRC-5 fibroblast cells were also reported by Radovanović et al. [47]. Furthermore, in their study, Thian et al. [44] showed that the incorporation of Zn^2+^ ions in the hydroxyapatite matrix could enhance the bioactivity of HAp. The study reported by the present supports our findings and highlights that zinc-doped hydroxyapatite (ZnHAp) exhibits good biocompatibility and could support cell viability and proliferation for numerous cell types, including human osteoblasts, mesenchymal stem cells, and fibroblasts. Furthermore, the results of the MTT assay also emphasized that ZnHApTe did not exhibit any toxic effects on the hFOB 1.19 cells for any tested time interval. The results showed a lower cellular viability than in the case of ZnHAp but still above 92%. In addition, these data also emphasized that the cellular viability of the hFOB 1.19 cells increased with an increase in the incubation time, reaching 94% after 72 h. These results emphasized that the presence of a small amount of tetracycline (5%) in the ZnHAp sample did not induce any cytotoxic effects. These results agree with previously reported data on the toxicity of tetracycline [50,51,52,53]. Tetracycline has been reported to exhibit good biological properties in small concentrations. In addition, studies have shown that tetracycline supports osteoblast function, including promoting proliferation, differentiation, and mineralization [53], while providing anti-inflammatory [54] and antioxidant benefits [55], creating a favorable environment for osteoblast function. However, the reported studies stress that insightful consideration should be attributed to the correct dosage necessary to avoid cytotoxic effects. Due to its broad-spectrum action and enhanced biological properties, tetracycline has been utilized in the development of novel compounds with biomedical applications. In their study regarding “controlled release and antibacterial activity of tetracycline hydrochloride-loaded bacterial cellulose composite membranes”, Shao et al. [52] reported that the BC-TCH composite films exhibited good biocompatibility and present effective antibacterial activity. Their results highlighted that TCH did not inhibit the proliferation of HEK293 cells, even at a high concentration [52]. Similar results were obtained by Dayaghi et al. [51], who reported that the presence of tetracycline in the composition of magnesium–zinc scaffolds guaranteed their antimicrobial character while exhibiting good biocompatible properties at small concentrations. Their study revealed that only the scaffolds with tetracycline concentrations of 1% and 5% were biocompatible, whereas the ones possessing higher dosages of tetracycline concentration demonstrated toxicity [51].

**Figure 12:**
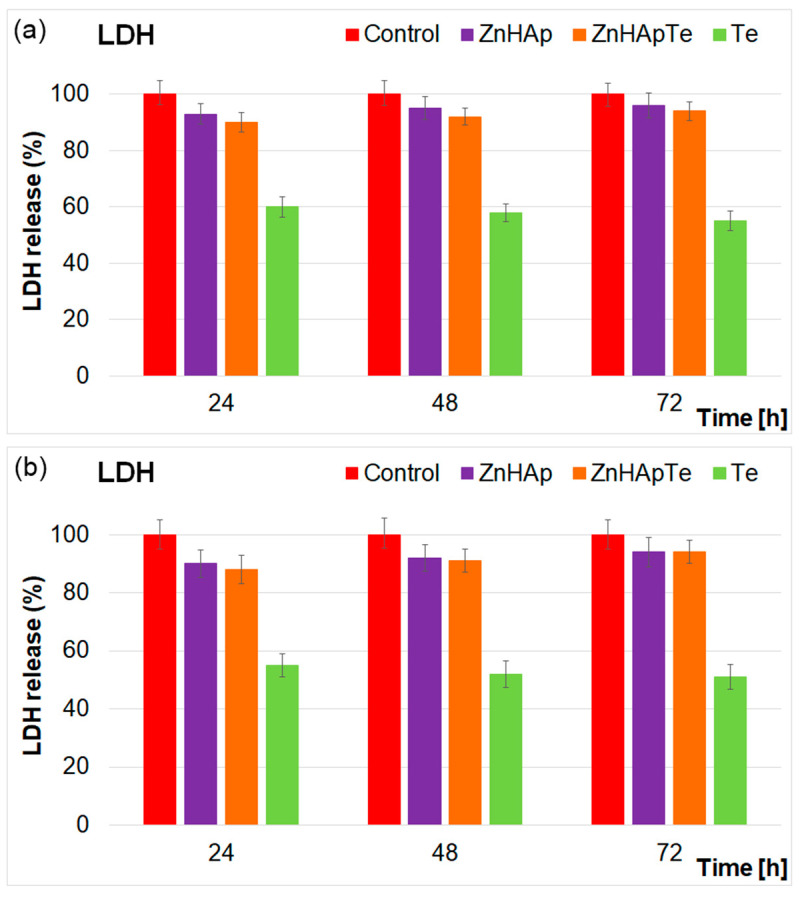
Lactate dehydrogenase (LDH) activity released in the culture medium of hFOB 1.19 cells after the treatment with 50 µg/mL (**a**) and 200 µg/mL (**b**) of ZnHAp, ZnHApTe, and Te for 24, 48 and 72 h. The results are represented as mean ± standard deviation (SD).

Complementary information regarding the cytotoxicity of the ZnHAp and ZnHApTe was obtained with the aid of Lactate dehydrogenase (LDH) release studies (Figure 12a,b).

The LDH assay is usually used to determine cell cytotoxicity by determining the release of LDH from the damaged cells into the culture medium. In evaluating the cytotoxicity of materials, this investigation can effectively assess the tested materials’ impact on cell viability. A low value of LDH release indicates that the tested material exhibits low cytotoxicity, suggesting that the cells remain viable and almost undamaged. On the other hand, a high LDH release is a sign of high cytotoxicity, indicating that the tested materials could cause substantial cell damage and significantly reduce the cell’s viability. The results of the LDH assay for the ZnHAp, ZnHApTe, and Te nanoparticles at different concentrations are depicted in Figure 12a,b. LDH activity was quantified in the supernatant of the cells to test the cytotoxicity and membrane integrity. These data were represented graphically as mean ± SD relative to the control sample, for which the LDH level was set as 100%. The results of the LDH assay emphasized that there were no statistically significant changes from the control for any of the analyzed samples. These findings indicate that exposure to the ZnHAp and ZnHApTe nanoparticles did not damage the hFOB 1.19 cell’s membrane, preserving its integrity. The result also indicates the absence of cell necrosis. The results from the LDH activity assay agreed with those obtained from the MTT assays, demonstrating that both the ZnHAp and ZnHApTe nanoparticles exhibited no cytotoxic activity against hFOB 1.19 cells. Data from both assays indicated that these nanoparticles possess good biocompatibility towards hFOB 1.19 cells. These findings emphasized that the ZnHAp and ZnHApTe nanoparticles are promising candidates for future development of biomaterials for biomedical applications.

**Figure 13:**
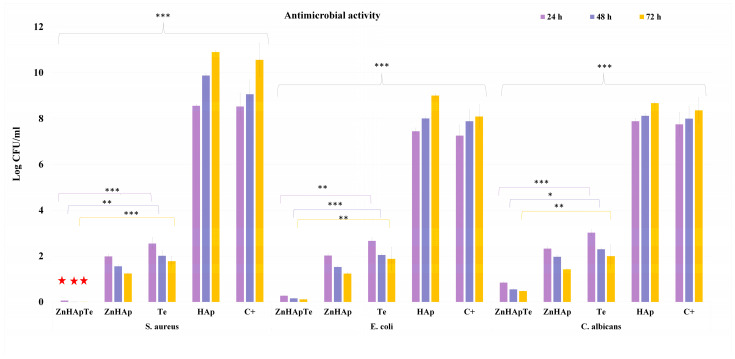
Graphical representation of the logarithmic values of colony forming units (CFU)/mL of *S. aureus* ATCC 25923, *E. coli* ATCC 25922, and *C. albicans* ATCC 10231 microbial strains after 24, 48, and 72 h of exposure to HAp, Te, ZnHAp and ZnHApTe. The results are presented as mean ± standard error. The statistical analysis was performed by ordinary one-way ANOVA. The *p*-values indicated are * *p* ≤ 0.002, ** *p* ≤ 0.001, *** *p* ≤ 0.0001. The red stars highlight the bactericidal effects of the samples.

Tetracycline is a well-known broad-spectrum antibiotic, which was discovered in the late 1940s and has been a fundamental tool in combating various bacterial infections since then. Its use is widespread because of its efficacy against diverse types of microorganisms, such as Gram-positive and Gram-negative bacteria, atypical pathogens, and some protozoa. The understanding of the antimicrobial range and the action mechanism of tetracycline is crucial for recognizing its clinical value and trying to resolve the emerging issue of antibiotic resistance at a global scale. In this context, the development of novel antimicrobial agents is of great significance worldwide. For this purpose, the antimicrobial effects of tetracycline-enriched ZnHAp were evaluated at three different time intervals using the most common microbial strains, *S. aureus*, *E. coli*, and *C. albicans*. The antimicrobial studies performed on the HAp, Te, ZnHAp, and ZnHApTe nanoparticles revealed that loading ZnHAp with tetracycline determined a complete bactericidal effect against *S. aureus*, increased the bacteriostatic activity against *E. coli*, and enhanced the antifungal activity against *C. albicans* (Figure 13). Furthermore, the studies presented in this paper have demonstrated that *S. aureus* exhibited greater sensitivity to the ZnHAp and ZnHApTe nanoparticles than *E. coli* ATCC 25922 and *C. albicans* ATCC 10231 compared with the control and pure tetracycline. The results of the in vitro antimicrobial assay depicted that HAp nanoparticles did not inhibit the development of any of the tested microbial strains for any given interval. In addition, these data emphasized that the HAp nanoparticles aided the development and proliferation of the microbial cells compared with the control. The results showed a significant increase in the CFU values of the microbial cells exposed to Hap nanoparticles compared with the control.

In addition, the results of the in vitro antimicrobial assays have demonstrated that the presence of tetracycline conferred the ZnHApTe biocomposite bactericidal properties against the *S. aureus* bacterial strain.

These results align well with previously reported studies on the antimicrobial effects of tetracycline [56,57,58,59]. The findings revealed that the antimicrobial activities of ZnHAp and ZnHApTe are also correlated with the incubation time. The results showed that even though the CFU values are low, even from the early development stage, the CFU values decreased significantly with the incubation time. The antimicrobial activity of the nanoparticles is attributed to both zinc ions from the hydroxyapatite matrix and the presence of tetracycline. Zinc ions (Zn^2+^) are well known to possess antimicrobial properties through multiple mechanisms. Zinc ions can compromise the cell’s membrane integrity by binding to its negatively charged components, thus increasing its permeability and, in the end, causing cell lysis. They could also inhibit the enzymatic activity by attaching themselves to active sites or by displacing some of the essential metal cofactors and disrupting the critical metabolic functions of the microbial cells. In addition, zinc ions have the ability to inhibit nutrient uptake by competing with the essential metal ions, leading to nutrient deprivation of the microbial cell. On the other hand, zinc ions could generate reactive oxygen species (ROS) that are responsible for inducing oxidative damage to DNA, proteins, and lipids. Zn^2+^ interferes with the processes of DNA replication and transcription by binding to the nucleic acids and other proteins, thus disrupting protein synthesis by interacting with the cell’s ribosomal components. Additionally, zinc could enhance the host immune response, aiding in infection clearance [60,61,62,63]. These mechanisms are reported to be responsible for zinc ions’ effectiveness in various antimicrobial applications, from medical treatments to food preservation [60,61,62,63,64,65,66,67]. On the other hand, it was reported that tetracycline’s antimicrobial properties could be attributed to its ability to inhibit protein synthesis in bacteria [68,69,70,71,72,73]. Tetracycline could also bind to the 30S ribosomal subunit, blocking the attachment of aminoacyl-tRNA to the mRNA-ribosome complex, thereby blocking the addition of new amino acids to the peptide chain and interrupting protein synthesis. This disruption could inhibit bacterial growth and replication. Additionally, tetracycline can compromise the integrity of the bacterial cell membrane. In addition, its broad-spectrum activity makes tetracycline effective against a wide range of Gram-positive and Gram-negative bacteria, as well as some intracellular pathogens. Its ability to pass through the bacterial cells and its bacteriostatic nature, which prevents bacterial multiplication without necessarily killing them, make it a valuable antibiotic for treating numerous infections [68,69,70,71,72,73]. In this context, the findings obtained from the antimicrobial assays that highlighted that zinc-doped hydroxyapatite enriched with tetracycline demonstrates enhanced antimicrobial properties by combining the effects of zinc ions and tetracycline agree with the existing studies. The combined effects of zinc ions could disrupt microbial cell membranes by binding to negatively charged components, increasing permeability that could cause cell lysis and could inhibit enzymatic activity and generate reactive oxygen species (ROS) and tetracycline, which has the ability to inhibit protein synthesis by binding to the 30S ribosomal subunit, preventing the attachment of aminoacyl-tRNA to the mRNA-ribosome complex, thus blocking the addition of new amino acids and preventing bacterial growth. The synergy between zinc’s numerous antimicrobial mechanisms and tetracycline’s specific antimicrobial functions contribute considerably to the fact that zinc-doped hydroxyapatite enriched with tetracycline is particularly effective against a wide range of bacteria, including resistant strains, enhancing its use in medical treatments and implants. The results of the in vitro antimicrobial assay highlighted that the synergy between the zinc ions from the HAp matrix, as well as the presence of tetracycline, leads to bactericidal properties against *S. aureus* and confers an increase in the bacteriostatic properties against *E. coli* to the ZnHApTe samples compared with simple ZnHAp and Te samples. In addition, a notable increase in the antifungal activity of the ZnHApTe samples was observed compared with the ZnHAp and Te samples. The findings obtained in this study suggest that the ZnHAp and ZnHApTe nanoparticles could be effectively used to develop novel antimicrobial agents.

## 3. Materials and Methods

### 3.1. Materials

For the development of zinc-doped hydroxyapatite (ZnHAp), zinc-doped hydroxyapatite enriched with tetracycline (ZnHApTe) powders were used the next reagents: calcium nitrate (Ca(NO_3_)_2_·4H_2_O), diammonium hydrogen phosphate ((NH_4_)_2_HPO_4_) and zinc nitrate (Zn(NO_3_)_6_·6H_2_O) and tetracycline hydrochloride (Te, C_22_H_24_N_2_O_8_·HCl).

### 3.2. Development of Zinc-Doped Hydroxyapatite Enriched with Tetracycline

The zinc-doped hydroxyapatite (ZnHAp) and zinc-doped hydroxyapatite enriched with tetracycline (ZnHApTe) powders were obtained through an adapted co-precipitation method. During the synthesis process, the ratio of [Ca + Zn]/P was equal to 1.67, and the zinc concentration was x_Zn_ = 0.1 [74]. The tetracycline solution, together with the zinc, calcium, and phosphate solution, was stirred well for 24 h under ambient conditions. Then, the phosphate solution was added to the calcium and zinc solution and stirred for 72 h. The final concentration of Te in ZnHApTe was 5%. The final mixture was centrifuged, and the resulting precipitate was washed five times with water. After the last centrifugation, the final precipitate was dried in air.

### 3.3. X-ray Diffraction (XRD)

Information regarding the structure of the obtained samples, including zinc-doped hydroxyapatite (ZnHAp), zinc-doped hydroxyapatite enriched with tetracycline (ZnHApTe), and tetracycline (Te), was achieved using X-ray diffraction (XRD). Measurements were conducted with a Bruker D8 Advance diffractometer, utilizing CuKα radiation (λ = 1.5418 Å) (Bruker, Karlsruhe, Germany) and equipped with a LynxEye™ 1D high-efficiency linear detector. Data were collected in the 2θ range of 10–60° with a step size of 0.02° and a time of 5 s per step.

### 3.4. Fourier Transform Infrared Spectroscopy (FTIR)

Fourier evaluated the functional groups present in the ZnHAp, Te, and ZnHApTe samples transform infrared spectroscopy (FTIR). The FTIR spectra were recorded with a Perkin Elmer spectrometer operated in ATR mode (attenuated total reflectance) using a Universal Diamond/KRS-5 (Perkin Elmer, Waltham, MA, USA) within the range of 450–4000 cm^−1^. The second derivative spectra were obtained after applying a 5-point smoothing of the original FTIR. The procedure followed for obtaining the deconvoluted spectra in the 900–1200 cm^−1^ spectral region was presented in detail in [75].

### 3.5. X-ray Photoelectron Spectroscopy (XPS)

The XPS analysis of ZnHAp, tetracycline, and ZnHApTe was performed using an X-ray photoelectron spectroscopy (XPS) investigation was conducted using an SES 2002 instrument from Scienta Omicron (Scienta Omicron, Taunusstein, Germany). A monochromatic Al K(alpha) X-ray source with an energy of 1486.6 eV was used. The analysis protocols, as well as the scanning parameters, were used in accordance with previous studies [76]. The CasaXPS 2.3.14 software (utilizing the Shirley background type) was employed [77]. All binding energy (BE) values presented in this study were adjusted to the C1s peak at 284.8 eV for charge correction.

### 3.6. Transmission Electron Microscopy (TEM)

For the TEM studies, a CM 20 (Philips FEI, Eindhoven, The Netherlands) transmission electron microscope (TEM). The TEM microscope was equipped with a Lab6 instrument.

### 3.7. Scanning Electron Microscopy (SEM)

The morphology evaluation of the ZnHAp and ZnHApTe nanoparticles was achieved using a scanning electron microscope (SEM, FEI Quanta Inspect F, FEI Company, Hillsboro, OR, USA). Moreover, the SEM microscope was equipped with an energy-dispersive X-ray (EDX) attachment to evaluate the chemical composition of the ZnHAp, Te, and ZnHApTe samples. Prior to the SEM examination, the samples were added to a carbon tape. The mean particle size estimation was performed by numbering approximately 500 nanoparticles.

### 3.8. Hemolysis Assay

The biological properties of the ZnHAp and ZnHApTe nanoparticles and Te powder were evaluated with the aid of a hemolysis assay. The experiments were performed using sheep red blood cells (RBCs) following the method described by Das et al. [78] modified as previously reported in [79]. For this purpose, 500 μL of samples of various concentrations in 0.9% NaCl were mixed with 500 μL of erythrocyte suspension. The tubes were mixed and incubated at 37 °C for 30 min. Equal amounts of erythrocyte suspension were added to Triton X-100 (Thermo Fisher Scientific, Waltham, MA, USA) and PBS for the positive and negative controls. After 30 min of incubation, the samples were centrifuged, and the supernatant was carefully transferred to 96-well plates. The absorbance of the supernatant was measured at 540 nm using a FlexStation 3 UV-VIS spectrophotometer (Molecular Devices Company, Sunnyvale, CA, USA) instrument. The hemolytic index (HI%) was calculated using the following equation:

(1) $$\mathrm{Hemolysis}\;(\%)=\frac{V_{OD\;sample}-V_{OD\;negative\;control}}{V_{OD\;positive\;control}-V_{OD\;negative\;control}}\times 100$$

### 3.9. MTT Assay

The cytotoxicity of the ZnHAp, ZnHApTe, and Te nanoparticles was assessed with the aid of human fetal osteoblastic cells (hFOB 1.19 cell line) using a methodology previously described in [79]. For this study, the hFOB 1.19 cells were cultured in Dulbecco Modified Eagle’s Medium at 37 °C in a humidified atmosphere with a CO_2_ concentration of 5%. The cells were seeded at a density of 3 × 10^4^ cells/cm^2^. The cell viability was determined using the colorimetric test assay 3-(4,5-dimethylthiazol-2-yl)-2,5-diphenyltetrazolium bromide (MTT; Sigma-Aldrich, St. Louis, MO, USA) assay. The viability was evaluated at three different time intervals of incubation, 24, 48, and 72 h, and for two different concentrations of the ZnHAp, ZnHApTe, and Te samples (50 and 200 µg/mL). After each incubation period, the medium was removed, and the cells were incubated using 1 mg/mL MTT and kept for 4 h at 37 °C. The cell viability was quantified based on the absorbance values measured at 595 nm using a microplate reader (Flex Station 3, Molecular Devices, San Jose, CA, USA). The percentage of viable cells was determined relative to the cell viability of the control sample, which was set to 100% viability, and the results were presented graphically as mean ± SD.

### 3.10. Lactate Dehydrogenase (LDH) Release Measurement

After the three different incubation periods of growth of human fetal osteoblastic cells (hFOB 1.19 cell line), the culture medium was collected, and LDH release was measured using the Cytotoxicity Detection KitPLUS (Roche, Mannheim, Germany) according to the manufacturer’s instructions. For this purpose, 50 µL of culture supernatants were mixed with 50 µL of reaction mixture containing the catalyst and dye solution and were incubated for 30 min in the dark. The absorbance was measured at a 485 nm wavelength using a Tecan GENios instrument.

### 3.11. In Vitro Antimicrobial Activity

The antimicrobial properties of the HAp, ZnHAp, ZnHApTe, and Te nanoparticles were assessed in vitro using one of the most common reference strains, *Staphylococcus aureus* ATCC 25923, *Escherichia coli* ATCC 25922, and *Candida albicans* ATCC 10231 (all from ATCC, Old Town Manassas, VA, USA). The antimicrobial assays followed previously reported methodologies [17] with 0.5 McFarland standard microbial cultures. The samples were inoculated with 1.5 mL microbial suspensions at a density of 5 × 10^6^ CFU/mL, prepared in phosphate-buffered saline (PBS), and incubated for 24, 48, and 72 h. Free microbial cultures served as positive controls (C+). Suspensions were collected at 24, 48, and 72 h, then incubated on LB agar medium for 24 h at 37 °C. The CFU/mL count was determined for each incubated sample. Experiments were performed in triplicate, and data were presented as mean ± SD. Statistical analysis was conducted using the ANOVA single-factor test.

## 4. Conclusions

The zinc-doped hydroxyapatite enriched with tetracycline (ZnHApTe) powders were obtained for the first time using an adapted coprecipitation method. The results of the XRD studies reveal the presence of the hydroxyapatite and Te in the ZnHApTe sample. The XRD results also underlined the absence of the additional phases. The presence of the functional groups that are characteristics of the HAp and Te structure in the ZnHApTe was highlighted by the FTIR results. The results of the XPS and EDS studies proved the purity of the samples. The antimicrobial assays revealed that both ZnHAp and ZnHApTe nanoparticles exhibited strong inhibitory effects on all the tested microbial strains for all the tested incubation intervals. Furthermore, the results also emphasized that the addition of tetracycline to ZnHAp nanoparticles considerably improved their antimicrobial activity, conferring them bactericidal properties against *S. aureus*, enhanced bacteriostatic activity against *E. coli* and better antifungal properties towards *C. albicans*. These data indicated that the antimicrobial activity was influenced by the incubation time and by the specific type of microbial strain.

The biological assays demonstrated that the ZnHAp and ZnHApTe nanoparticles exhibited good biocompatibility and antimicrobial properties. The hemolysis assay highlighted that the ZnHAp and ZnHApTe nanoparticles had a hemolytic index below 1%, which ensures that they are promising materials for being used in biomedical applications such as bone grafts, dental implants and tissue engineering scaffolds. Furthermore, the cell viability studies depicted that hFOB 1.19 cells maintained cellular viability higher than 94% in the presence of ZnHAp nanoparticles and above 92% in the presence of the ZnHApTe nanoparticles. Moreover, the results have emphasized that the cellular viability of hFOB 1.19 cells increased with an increase in the incubation time. In addition, the LDH assays also showed that exposure to the ZnHAp and ZnHApTe nanoparticles did not damage the hFOB 1.19 cell’s membrane, preserving its integrity. The findings of the biological assays depict the potential of the ZnHAp and ZnHApTe nanoparticles to be used in the future for the development of novel biomaterials with biomedical applications.

## Figures and Tables

**Table 1:** The results of EDS quantitative analyses performed on ZnHAp and ZnHApTe.

Sample	Ca	P	O	Zn	N	Cl
ZnHAp	16.9	10.68	71.49	0.93	-	-
ZnHApTe	20.18	12.65	51.6	0.92	0.95	13.7

## Data Availability

The original contributions presented in the study are included in the article; further inquiries can be directed to the corresponding authors.
